# Genetic Heterogeneity in Renal Cell Carcinoma: A Case of Concurrent Clear Cell and Papillary Renal Carcinomas

**DOI:** 10.1155/criu/5538985

**Published:** 2026-10-02

**Authors:** Annelies Kerckhofs, Stéphanie Dautricourt, Léon van Kempen, Stefan De Wachter, Ali Ramadhan

**Affiliations:** ^1^ Department of Pathology Antwerp University Hospital Edegem Belgium uza.be; ^2^ Department of Urology Antwerp University Hospital Edegem Belgium uza.be; ^3^ Department of Urology Antwerp Surgical Training Anatomy and Research Centre (ASTARC) Faculty of Medicine and Health Sciences University of Antwerp Wilrijk Belgium uantwerpen.be

**Keywords:** adrenal metastasis, clear cell RCC, molecular profiling, next-generation sequencing (NGS), papillary RCC, renal cell carcinoma, synchronous tumors, tumor clonality

## Abstract

**Background/Objectives:**

Recent advancements in genetic research have significantly enhanced the understanding of renal cell carcinoma (RCC), now recognized as a heterogeneous group with distinct genetic features. This case report is aimed at illustrating its genetic diversity and implications for diagnosis and treatment.

**Methods:**

A middle‐aged man presented with an incidental finding of RCC in the right kidney on computer tomography (CT) performed for a persistent cough. Comprehensive radiological and blood tests were conducted, followed by a total right nephrectomy. Histological examination showed a concurrent appearance of two manifestly different tumors in the kidney. Molecular analyses, including SNP array and next‐generation sequencing (NGS), were conducted to identify genetic mutations and assess clonality.

**Results:**

The nephrectomy identified two distinct tumors: a clear cell RCC (ISUP/WHO grade 3, pT1b) and a papillary RCC (ISUP/WHO grade 2, pT1a). Molecular analysis confirmed *VHL*, *TP53*, and *SETD2* mutations in the clear cell RCC, whereas no mutations were detected in the papillary RCC. The SNP array revealed significant chromosomal differences, suggesting the tumors were not clonally related. Follow‐up imaging detected an adrenal metastasis from the clear cell RCC. The metastasis showed a *VHL* and *SETD2* mutation, which undoubtedly proved to be derived from the clear cell RCC.

**Conclusions:**

This case underscores the genetic heterogeneity of RCC and the importance of molecular profiling in guiding diagnosis and treatment. The distinct genetic profiles of the two tumors suggest they are not clonally related, highlighting the complexity of RCC and the need for individualized therapeutic approaches.

## 1. Introduction

Renal cell carcinoma (RCC) is a heterogeneous group of malignancies arising from the renal parenchyma, accounting for approximately 3%–4% of all adult cancers globally. Among the subtypes, clear cell renal cell carcinoma (ccRCC) is the most prevalent, representing 75%–80% of cases, whereas papillary renal cell carcinoma (pRCC) accounts for 10%–15% and chromophobe subtypes for 5%. These subtypes are distinguished by unique histopathological and molecular features, which have significant implications for their clinical behavior, prognosis, and treatment response [1, 2]. Occurrence of distinct RCC subtypes in the same kidney is exceedingly rare, with limited cases reported in literature, sometimes presenting in a syndromic setting [3–5].

Here, we report the case of a 60‐year‐old man with unilateral synchronous ccRCC and pRCC in the right kidney, further complicated by an adrenal metastasis. This case emphasizes the clinical and pathological challenges in diagnosing and managing multiple RCC subtypes within a single patient. The inclusion of comprehensive histopathological, immunohistochemical, and molecular analyses was pivotal in characterizing the tumors and confirming their independent origins.

To our knowledge, this is one of the few cases documented in the literature that provides detailed molecular profiling of synchronous ccRCC and pRCC, along with an analysis of the metastatic lesion [6, 7]. This report contributes to the growing body of evidence on the heterogeneity of RCC and highlights the importance of integrating molecular diagnostics into clinical practice. By delineating the distinct genetic landscapes of ccRCC and pRCC, this study reinforces the need for subtype‐specific therapeutic strategies, especially in the era of precision oncology.

## 2. Case Presentation

A 60‐year‐old man presented to the urological department after an incidental radiological finding of a renal mass on computer tomography (CT) thorax. The scan, initially performed due to a persistent cough, showed apart from nonspecific findings in the lungs, a mass in the right kidney.

A complete diagnostic work‐up, including radiological imaging and blood tests, was performed. A CT abdomen demonstrated a nonhomogeneous suspicious mass in the upper pole of the right kidney with a maximal diameter of around 7 cm. The lesion consisted of partly cystic‐necrotic as well as solid components, extending into the renal pelvis. Additionally, a second lesion was detected, more specifically a millimetric cyst in the interpolar region of the right kidney. This was radiologically suspicious for a benign hemorrhagic cyst (Figure 1).

**Figure 1:**
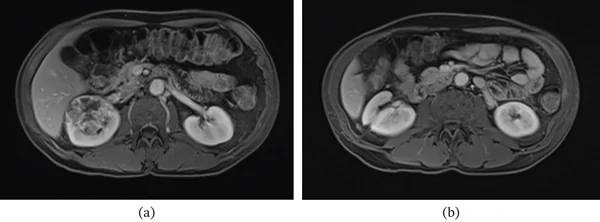
CT abdomen on the level of the kidneys. In Panel (a), the partly cystic, solid, and hemorrhagic lesion is seen in the right kidney. In Panel (b), the smaller, more subcapsular lesion is seen also in the right kidney. On this CT image, this second lesion is suspected to be a cyst.

The patient underwent a total right nephrectomy which macroscopically showed two prominent lesions (Figure 2). The first tumor, located in the upper pole, measured approximately 6.5 cm in maximal diameter. This tumor was nonhomogeneous, with a yellow to orange appearance and cystic components. No apparent breakthrough through the kidney capsule and no apparent involvement of the renal pelvis or renal vessels were noted. Despite the large tumor size and gross appearance of some outpouchings, definite renal sinus/vein branch invasion could not be confirmed. The second lesion was found subcapsular in the lower pole with a maximal diameter of 1.2 cm. There was a distinct distance and tumor‐free zone between these two tumors.

**Figure 2:**
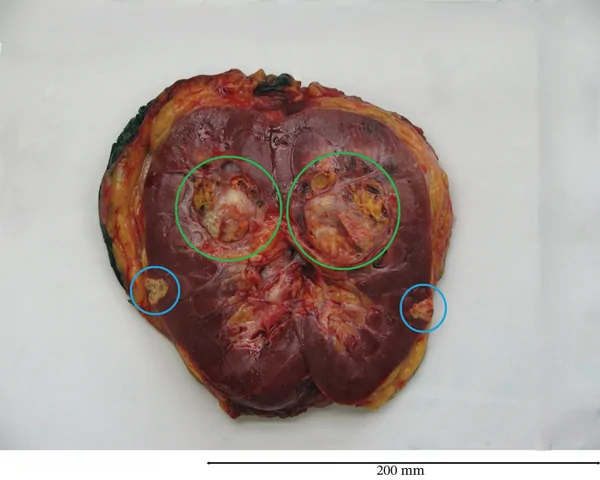
Macroscopical view of the bivalved right kidney. The two tumors are marked. The first tumor is seen in the upper pole, rather large and heterogeneous (green circle). The second lesion is seen interpolar and more subcapsular with a solid look (blue circle).

Histological examination confirmed the presence of two separate tumors. The first tumor showed a prominent clear cell pattern with an ISUP/WHO grade 3. The tumor showed cystic differentiation and necrosis. There was no evidence of perineural, lymphatic or perivascular invasion. A pathological TNM‐staging was suggested as a pT1b. (Figure 3) The second tumor showed a completely different architecture, featuring a papillary pattern without any clear cell component. The cells showed cytological atypia with an ISUP/WHO grade 2. There was no necrosis, nor any rhabdoid or sarcomatous morphology seen. This tumor was given another suggestion for TNM‐staging, more specifically pT1a. (Figure 4) Both tumors exhibited completely different histological characteristics, as well as distinct immunohistochemical patterns. The first tumor was strongly positive for CA9 and CD10, whereas negative for AMACR, keratin 7, keratin 20, and CD117. In contrast, the second tumor was negative for CA9 and CD10 but strongly positive for keratin 7 and AMACR.

**Figure 3:**
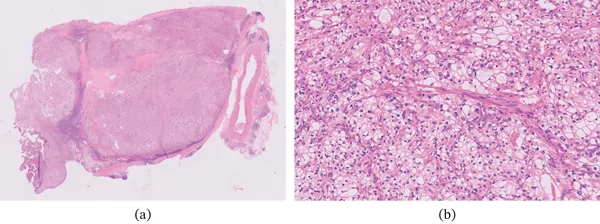
Histological image of the largest tumor. (a) At a low magnification, a prominent heterogeneous tumor with necrosis and hemorrhage is seen. (b) When looking closer, there is a prominent clear cell pattern with nucleoli clearly visible at a 100x magnification (ISUP/WHO grade 3).

**Figure 4:**
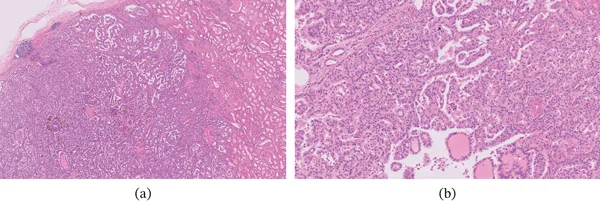
(a,b) Histological image of the smaller tumor, interpolar tumor. (a) The tumor shows an infiltrative growth pattern with a papillary aspect. The tumoral cells are more monotonous in comparison with the first tumor with nucleoli only visible at a 400x magnification (ISUP/WHO grade 2).

Based on the combined histomorphological and immunohistochemical findings, the first tumor was diagnosed as ccRCC, whereas the second was ultimately classified as pRCC. Although the lesion did not strictly fulfill the conventional WHO size criterion for papillary RCC, the absence of a distinct fibrous capsule and the presence of focal higher grade morphological features favored this diagnosis over papillary adenoma.

During a follow‐up radiological investigation 3 months later, an adrenal lesion was identified. Histological examination of the lesion revealed a metastasis of the ccRCC with an immunohistochemical profile identical to the largest primary tumor (CA9 and CD10 strongly positive, keratin 7, CD117 [KIT], and AMACR completely negative).

Because of the rarity of this case, a full molecular workup was performed. Single nucleotide polymorphism (SNP) array analysis on both tumors showed numerous copy number variations (CNVs) in the clear cell component, complicating the determination of copy neutral loss of heterozygosity (CN‐LOH). The SNP array demonstrated major chromosomal differences between the two tumors, suggesting that they are likely not clonally related. This can be nicely seen in Figure 5.

**Figure 5:**
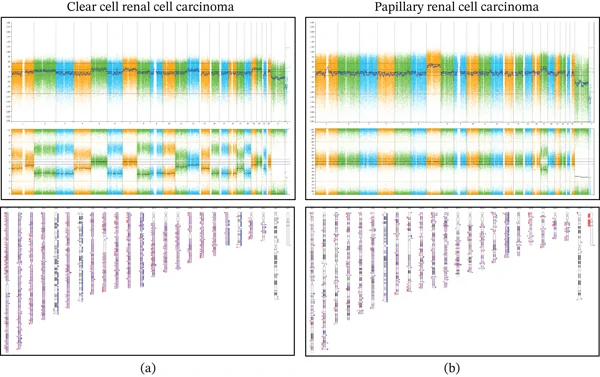
SNP array analysis of both tumors. In Panel (a), the CNV′s and the biallelic frequency (BAF) of the clear cell RCC is shown. The numerous CNV′s of this tumor are markedly seen. In Panel (b), side the CNV′s of the papillary RCC show far less variation with clear alterations in Chromosomes 7, 17, and Y.

Next‐generation sequencing (NGS) revealed the characteristic genetic signature of ccRCC, including pathogenic mutations in *VHL*, *TP53* and *SETD2*. On the other hand, no significant mutations were detected in the pRCC (Figure 6).

**Figure 6:**
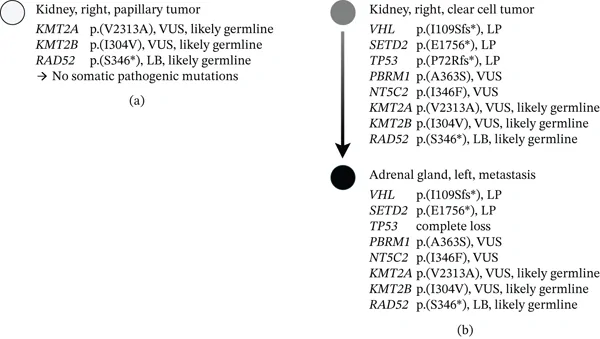
NGS analysis of both tumors showing no pathogenic mutations found in the (a) papillary RCC and (b) likely pathogenic mutations in the *VHL*, *SETD2,* and *TP53* in the clear cell RCC. On the bottom of Panel (b), the mutations found in the adrenal gland metastasis are shown, namely the *VHL* and *SETD2* mutations.

The adrenal gland metastasis was also subjected to a molecular investigation. The *VHL* mutation as well as the *SETD2* mutation were identified, whereas allelic inactivation of *TP53* was detected. Therefore, the adrenal gland metastasis most likely originated from the clear cell renal tumor based on histological and molecular features.

## 3. Discussion

The findings in this case highlight the distinct biological and molecular identities of ccRCC and pRCC, supporting the notion that these tumors are separate entities rather than deriving from one another. Both tumors presented unique histological and molecular profiles that served as critical tools in distinguishing their origins and understanding their behavior. The molecular analysis of the adrenal metastasis confirmed its origin from the ccRCC, as evidenced by the presence of hallmark mutations and chromosomal aberrations typically seen in ccRCC.

On one hand, there is the ccRCC with a variable molecular profile to be known in literature. In up to 80 to 90% of cases of sporadic, nonfamilial ccRCC, somatic mutations in the *Von Hippel Lindau (VHL*) gene were identified [8, 9]. *VHL* has a role in the hypoxia‐inducible factor (HIF) pathway, which regulates the response to oxygen levels.

In ccRCC, SNP arrays frequently reveal loss of heterozygosity on Chromosome 3p, often associated with *VHL* gene inactivation, as well as other tumor suppressor genes such as *PBRM1* and *BAP1*. Additional chromosomal losses on 8p, 9p, and 14q and gains on 5q are also commonly observed [9, 10].

On the other hand, the other tumor found in this case was a pRCC whose molecular profile is profoundly different compared with that of ccRCC. This tumor is, compared with other renal cell neoplasms, more common in chronic renal disease and acquired cystic disease. A small group occurs in a setting of hereditary pRCC. These tumors present with a germline mutation of *MET* [11]. PRCC′s are molecularly rather heterogeneous with genetic studies showing typical (partial) loss of chromosome Y in more than 70% of pRCC cases and gains in Chromosomes 7 and 17. The loss of Chromosome Y is not that rare in renal cancer or any other kind of cancer, such as squamous cell carcinomas, gastric cancer or pancreatic neoplasms. The occurrence is also described in oncocytomas, chromophobe carcinomas and ccRCCs [12].

Unlike ccRCC, PRCC does not have specific abnormalities in the *VHL* gene. PRCC′s resistance to *VHL*‐targeted therapies suggests an alternative pathway for HIF dysregulation. These molecular differences not only underscore the genetic heterogeneity of RCC subtypes but also explain their divergent responses to targeted therapies, such as those aimed at the HIF pathway, which are effective in ccRCC but not in pRCC.

The adrenal metastasis in this case aligns with the typical metastatic patterns observed in RCC, particularly ccRCC. Among renal tumors, ccRCC has a higher propensity to metastasize to the adrenal glands compared with other subtypes such as pRCC [13]. Molecular profiling of the adrenal lesion revealed mutations consistent with the ccRCC primary tumor, reinforcing its origin from the ccRCC rather than the pRCC. This is in agreement with studies showing that ccRCC frequently utilizes hematogenous and lymphatic routes to spread, with adrenal involvement observed in 6%–10% of cases. In contrast, adrenal metastases from pRCC are rare, accounting for only about 7% of its metastatic cases [13, 14].

## 4. Conclusions

The coexistence of a ccRCC and pRCC within the same kidney is rare and poses challenges in diagnosis and management. The understanding of RCC has significantly evolved, revealing distinct genetic pathways for different subtypes. The current study indicates independent simultaneous development of ccRCC and pRCC, characterized by their unique genetic fingerprints and excludes morphological diversity of one and the same tumor. The identification of shared mutations between the ccRCC and the adrenal metastasis highlights the clonal evolution typical of ccRCC, which is associated with a more aggressive metastatic behavior compared with pRCC. Accurate determination of the metastatic origin not only aids in understanding the tumor′s biology but also has significant therapeutic implications, as treatment strategies for ccRCC differ from those for pRCC and other RCC subtypes.

**Nomenclature**
BAF: biallelic frequency
ccRCC: clear cell renal cell carcinoma
CN‐LOH: copy neutral loss of heterozygosity
CNV: copy number variation
LB: likely benign
LP: likely pathogenic
NGS: next‐generation sequencing
pRCC: papillary renal cell carcinoma
RCC: renal cell carcinoma
SNP: single nucleotide polymorphism
VUS: variant of unknown significance

## Author Contributions

The authors take full responsibility for this article.

## Funding

No funding was received for this manuscript.

## Disclosure

All authors have read and approved the final version of the manuscript. Dr. Annelies Kerckhofs had full access to all of the data in this study and takes complete responsibility for the integrity of the data and the accuracy of the data analysis.

## Consent

Written informed consent has been obtained from the patient to publish this paper.

## Conflicts of Interest

The authors declare no conflicts of interest.

## Supporting information


**Supporting Information** Additional supporting information can be found online in the Supporting Information section.

## Data Availability

The data that support the findings of this study are available from the corresponding authors upon reasonable request.
