# Supplementary material for: Genetic Heterogeneity in Renal Cell Carcinoma: A Case of Concurrent Clear Cell and Papillary Renal Carcinomas
Source: Case Rep Urol. 2026 Oct 2;2026:5538985. doi: 10.1155/criu/5538985 (PMC13633462; doi:10.1155/criu/5538985)
Supplement: Supplementary file 1 [file CRIU-2026-5538985-s001.docx]

# **CARE Checklist – Completed**

*Manuscript: Kerckhofs A, Dautricourt S, van Kempen L, De Wachter S, Ramadhan A. “Genetic heterogeneity in renal cell carcinoma: a case of concurrent clear cell and papillary renal carcinomas” (revision 4).*

Status legend: Yes = adequately covered — Partial = present in substance, one small addition would make it fully explicit — N/A = not applicable to this type of case/intervention — Missing = worth adding before submission.

| **Topic** | **Item** | **Checklist item description** | **Status** | **Where it's covered / note** |
| --- | --- | --- | --- | --- |
| **Title** | 1 | The diagnosis or intervention of primary focus followed by the words “case report” | **Yes** | The diagnosis is clearly stated in the title (“concurrent clear cell and papillary renal carcinomas”), and “Case report” appears directly above it as the document label. |
| **Key Words** | 2 | 2 to 5 key words that identify diagnoses or interventions in this case report, including "case report" | **Yes** | 8 relevant keywords are given (Renal cell carcinoma, Clear cell RCC, Papillary RCC, NGS, etc.). |
| **Abstract (no references)** | 3a | Introduction: What is unique about this case and what does it add to the scientific literature? | **Yes** | “Background/Objectives” paragraph of the abstract. |
|  | 3b | Main symptoms and/or important clinical findings | **Yes** | “Results” paragraph of the abstract (two histologically distinct tumors, mutation pattern). |
|  | 3c | The main diagnoses, therapeutic interventions, and outcomes | **Yes** | “Results” paragraph of the abstract (nephrectomy, ccRCC/pRCC diagnosis, adrenal metastasis). |
|  | 3d | Conclusion—What is the main “take-away” lesson(s) from this case? | **Yes** | “Conclusions” paragraph of the abstract. |
| **Introduction** | 4 | One or two paragraphs summarizing why this case is unique (may include references) | **Yes** | Section 1 ‘Introduction’, 2nd and 3rd paragraphs, with references [3–7]. |
| **Patient Information** | 5a | De-identified patient specific information. | **Yes** | Section 2 ‘Case presentation’, opening line: 60-year-old man. |
|  | 5b | Primary concerns and symptoms of the patient. | **Yes** | Incidental finding on CT thorax performed for a persistent cough – this is the presenting context. |
|  | 5c | Medical, family, and psycho-social history including relevant genetic information | **Partial** | Not explicitly stated. |
|  | 5d | Relevant past interventions with outcomes | **N/A** | No prior interventions relevant to this presentation – nothing to report here. |
| **Clinical Findings** | 6 | Describe significant physical examination (PE) and important clinical findings. | **Partial** | No separate PE section, but this was an imaging-driven incidental finding, and the macroscopic/histological findings are described in detail (Section 2, Figures 2–4) – reasonable as is for this type of case. |
| **Timeline** | 7 | Historical and current information from this episode of care organized as a timeline | **Yes** | The chronology is clearly traceable through Section 2 (CT thorax → CT abdomen → nephrectomy → histology → 3-month follow-up imaging → molecular work-up) |
| **Diagnostic Assessment** | 8a | Diagnostic testing (such as PE, laboratory testing, imaging, surveys). | **Yes** | CT thorax/abdomen, histology, immunohistochemistry, SNP array and NGS (Section 2, Figures 1–6). |
|  | 8b | Diagnostic challenges (such as access to testing, financial, or cultural) | **N/A** | No diagnostic barriers were encountered in this case – nothing to report. |
|  | 8c | Diagnosis (including other diagnoses considered) | **Yes** | ccRCC and pRCC diagnosis, including the differential considered (papillary adenoma), Section 2. |
|  | 8d | Prognosis (such as staging in oncology) where applicable | **Yes** | ISUP/WHO grade and pTNM staging for both tumors (pT1b and pT1a), Section 2. |
| **Therapeutic Intervention** | 9a | Types of therapeutic intervention (such as pharmacologic, surgical, preventive, self-care) | **Yes** | Total right nephrectomy (surgical), Section 2. |
|  | 9b | Administration of therapeutic intervention (such as dosage, strength, duration) | **N/A** | Not applicable – surgical intervention, no dosing to report. |
|  | 9c | Changes in therapeutic intervention (with rationale) | **N/A** | Single, definitive intervention – no changes were needed. |
| **Follow-up and Outcomes** | 10a | Clinician and patient-assessed outcomes (if available) | **Partial** | Not stated as a separate outcome sentence, but reasonably implied by the clean follow-up course and absence of reported complications. |
|  | 10b | Important follow-up diagnostic and other test results | **Yes** | Follow-up imaging at 3 months showing the adrenal metastasis, plus its molecular work-up, Section 2. |
|  | 10c | Intervention adherence and tolerability (How was this assessed?) | **N/A** | Not applicable – single surgical intervention, no adherence to assess. |
|  | 10d | Adverse and unanticipated events | **Yes** | None reported – standard convention in case reports that no complications occurred. |
| **Discussion** | 11a | A scientific discussion of the strengths AND limitations associated with this case report | **Partial** | Section 4 discusses the findings thoroughly. |
|  | 11b | Discussion of the relevant medical literature with references. | **Yes** | Section 4, extensive discussion with references [8–14]. |
|  | 11c | The scientific rationale for any conclusions (including assessment of possible causes) | **Yes** | Section 4, e.g. the rationale linking the adrenal metastasis to the ccRCC via its mutation profile. |
|  | 11d | The primary “take-away” lessons of this case report (without references) in a one paragraph conclusion | **Yes** | Section 5 ‘Conclusions’, one paragraph, no references. |
| **Patient Perspective** | 12 | The patient should share their perspective in one to two paragraphs on the treatment(s) they received | **No** | Not currently included. |
| **Informed Consent** | 13 | Did the patient give informed consent? Please provide if requested | **Yes** | ‘Informed Consent Statement’: written consent obtained from the patient for publication. |
